# A qualitative study of professional and carer perceptions of the threats to safe hospital discharge for stroke and hip fracture patients in the English National Health Service

**DOI:** 10.1186/s12913-016-1568-2

**Published:** 2016-07-25

**Authors:** Justin Waring, Simon Bishop, Fiona Marshall

**Affiliations:** Centre for Health Innovation, Leadership & Learning, Nottingham University Business School, Jubilee Campus, Nottingham, UK

**Keywords:** Patient safety, Hospital discharge, Stakeholder, Qualitative, UK

## Abstract

**Background:**

Hospital discharge is a vulnerable transitional stage in patient care. This qualitative study investigated the views of healthcare professionals and patients about the threats to safe hospital discharge with aim of identifying contributory and latent factors. The study was undertaken in two regional health and social care systems in the English National Health Service, each comprising three acute hospitals, community and primary care providers and municipal social care services. The study focused on the threats to safe discharge for hip fracture and stroke patients as exemplars of complex care transitions.

**Methods:**

A qualitative study involving narrative interviews with 213 representative stakeholders and professionals involved in discharge planning and care transition activities. Narratives were analysed in line with ‘systems’ thinking to identify proximal (active) and distal (latent) factors, and the relationships between them.

**Results:**

Three linked categories of commonly and consistently identified threat to safe discharge were identified: (1) ‘direct’ patient harms comprising falls, infection, sores and ulceration, medicines-related issues, and relapse; (2) proximal ‘contributing’ factors including completion of tests, assessment of patient, management of equipment and medicines, care plan, follow-up care and patient education; and distal ‘latent’ factors including discharge planning, referral processes, discharge timing, resources constraints, and organisational demands.

**Conclusion:**

From the perspective of stakeholders, the study elaborates the relationship between patient harms and systemic factors in the context of hospital discharge. It supports the importance of communication and collaboration across occupational and organisational boundaries, but also the challenges to supporting such communication with the inherent complexity of the care system.

## Background

Hospital discharge describes the point where patient care and recovery within an in-patient acute facility is transferred and continued within a community, social care or domestic setting. Policies repeatedly suggest hospital discharge is not an end-point in patient care, but rather one of multiple transitions within the patient’s journey [1, 2]. The organisation and provision of this *transitional care* typically involves multiple health and social care actors, who need to coordinate their specialist activities so patients receive integrated and, importantly, safe care. The problems of coordinating these various actors have resulted in hospital discharge being identified as a vulnerable or ‘at risk’ transitional stage. This vulnerability has been associated with system pressures, such as ‘delayed discharge’ or ‘bed blocking’ because of the failure to coordinate care [2–5]. According to Victor et al. [6], nearly 30 % of older people experience some delay in their hospital discharge, which exposes patients to additional hospital-related risks, creates emotional and physical dependency, incurs additional hospital costs and restricts the availability of hospital beds.

Studies show that care quality can be suboptimal during, or as a consequence of, hospital discharge [5]. Data provided by the former English National Patient Safety Agency (NPSA) [7] indicates that ‘*transfer/discharge of patient and infrastructure*’ accounted for 7–8 % of reported safety incidents in 2009. This figure is likely to be a significant underestimate given most incident reporting systems are not well utilised across health and social care boundaries. A major telephone survey of 400 patients following discharge found that nearly 20 % reported some form of adverse event, of which 6 % were preventable and 6 % ameliorable [8]. Research highlights a number of common discharge-related risks including the management of medicines, the provision of appropriate health and social care, incomplete tests and scans, the fitting and use of home adaptation, and the risks of falls, infections or sores [4, 5, 8–11]. The underlying sources of these risks can range from factors related to the patient condition or co-morbidities, to the assessment of patient need, the availability of specialist resources in the community and wider organisational factors. However, there is limited evidence of the common contributing factors that influence the safety of transitional care.

Of the factors often identified as contributing to unsafe discharge, communication is often seen as integral to the quality and safety of hospital discharge. Returning to NPSA data [7], ‘*notifying and organising external services*’ was identified as the most common category of reported incident. A systematic review conducted by Kripalani et al. [4] found that communications between hospitals and family doctors were often partial or missing, relying primarily upon frequently incomplete, ambiguous and delayed discharge summaries. A recent large-scale European study found consistent problems in communication and information exchange during discharge processes as contributed to common problems of medicines reconciliation, loss of information, and absence of care plans [12–14]. Furthermore, complications and risks are exacerbated by a failure to involve patients and families in the discharge process [12].

Significantly, problems with communication and coordination can be interpreted as stemming from the inherent complexity of the health and social care system, where there are multiple inter-dependent organisations and specialists involved in the discharge planning and care transition. Glasby [2] highlights three dimensions as influencing the interaction of different agencies: *occupational factors* related to the particular knowledge, cultures and practices of different professionals; *organisational factors* related to the working patterns, capabilities and resources of different agencies; and *compatibility and coordinating factors* related to how occupational and organisational factors are aligned, or differences reconciled. These represent wider systemic factors that influence the safety of care transitions.

Current thinking in the field of patient safety conceptualises the threats to safety as stemming not from individual negligence, malice or incompetence, alone, but as being brought about or made possible by the influence of ‘upstream’ factors located, for example, in the way groups work together, the design and management of work, and the wider organisation of care [15, 16]. This promotes analysis of the links between active errors and upstream latent factors. Although this line of thinking has been applied extensively to healthcare, the focus has typically remained *within* care domains, such as the ward or operating department. As a patient safety problem, hospital discharge is therefore significant because it highlights the potential for latent factors to be located *across* the wider care system at the inter-organisational relationships, thereby presenting more complex and challenging sources of risk.

Despite increased understanding of the types of risk patients can experience during hospital discharge, there remains only partial evidence about the interaction between proximal contributory factors, and distal latent factors. There is also limited understanding about how patients and staff involved in discharge planning and care transition perceive the relationship between more active and latent factors, and what this might tell us about stakeholders’ different perceptions and understanding of hospital discharge. This qualitative study investigated the views of healthcare professionals and patients and their carers about the threats to safe hospital discharge with aim of identifying contributory and latent factors.

## Methods

### Sampling and selection

The study was undertaken in two geographically distinct English care systems, equivalent to a local district or municipal authority, each comprising a single acute National Health Service (NHS) care provider with three hospital sites, various community and primary healthcare services, and social care services provided by statutory, private sector and third sector agencies. The systems differed in terms of their relative geographical size and population density, ethnic diversity and urban/rural balance; and also the configuration of the care systems including the size and scope of the acute care providers (e.g. teaching and research intensity). It was anticipated these differences might result in different threats to safety.

Within each care system the study focused on the discharge of stroke and hip fracture patients as two prominent examples of care transitions. Stroke is the third leading cause of death in the UK, the single largest cause of disability in community settings, where over 50 % of strokes result in some form of permanent disability and the annual cost to the NHS of providing stroke care is over £2.8bn [17]. Hip fracture is similarly a very common cause of hospital admission, making up around a quarter of the 310,000 patients who present to hospital with fractures annually in the UK, the majority of whom are over 65 [18]. Both stroke and hip fracture patients tend have multiple co-morbidities, including cognitive and physical impairment, which leads to longer lengths of stay, more complex discharge planning and higher readmission rates. These patient groups were therefore selected on the basis of being growing areas of acute hospital admission, but where on-going rehabilitation is increasingly provided outside of the hospital thereby requiring integration of health and social care services; and also because these patients both tend to be over 65 and able to represent the complexities associated with discharging older patients with complex care needs and co-morbidities.

For individual study participants, a purposive, snowball sampling approach was taken that aimed to recruit representatives from across the diversity of organisations and professionals involved in discharge planning and care transition of stroke and hip fracture patients within these two systems. In total 213 individuals were recruited and took part in the study. Sampling commenced within the stroke and hip fracture services for the main acute hospital for each care system. After securing access permissions, service leaders, medical, nursing and therapist and administrative leaders were invited in writing to take part in the study. Through consultation with these participants and taking a snowball approach, additional actors were progressively identified and invited to take part in the study. A parallel process was undertaken with community based hospitals, social care agencies and voluntary groups within each region, starting with designated service leaders who then identified additional participants. There were challenges in recruiting family doctors (General Practitioners (GPs)) to participate in the study, due to workload pressures, as such a separate focus group was organised with 5 GPs from each care system recruited through regional commissioning bodies. The study also invited patients, and their families, admitted to the stroke and hip fracture services of each acute hospital to participate in the study. Patients were identified in consultation with the clinical lead for each service, at the time when discharge planning was about to commence. Sampling also took into account differences in patient’s age, gender and ethnicity. Table 1 gives a full breakdown of study participants.

**Table 1:** Detail of study participants

Group	Site 1	Site 2	Total
Medical (hospital)	10	8	18
Nursing (hospital)	18	15	33
Health Care Assistants (HCAs)	5	2	7
Occupational Therapists	10	10	20
Physiotherapists	16	8	24
Other therapists (speech, dieticians)	2	3	5
Hospital Pharmacists	1	2	3
Ambulance (regional)	n/a	n/a	2
Administrative	2	2	4
Managerial/Leadership	3	3	6
Social Work	9	5	14
Social Care	2	2	4
Community Nursing	2	7	9
General Practitioners (GP)	1	2	3
GP administration	2	0	2
Support group/voluntary	4	2	6
Patients	16	14	30
Carers/Family	12	11	23

The study received ethical approval in May 2011 from NRES Committee East Midlands: Nottingham 1. The study also received R&D Approval from each participating NHS Trust, including letters of access for all researchers. All participants provided written consent prior to taking part in the study. The authors declare that they have no competing interests.

### Data collection

**Fig. 1:**
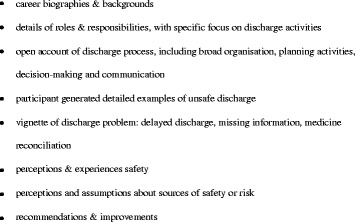
Interview topic guide

The study was carried out between September 2011 and March 2013. Data was collected through semi-structured qualitative interviews with each participant. The use of interview methods in patient safety research is widely accepted as useful for understanding how individuals and groups perceive, experience and give meaning to safety threats. Cognitive interviewing techniques are often favoured for this type of research resulting in ‘eye witness’ testimonies. Although there are clear merits in using this approach when investigating specific incidents, this study was more concerned with exploring the broader experiences, perceptions and assumptions of different stakeholders. As such, the interviews followed a narrative approach [19] that encouraged participants to develop rich accounts or stories of the discharge process, as a way of identifying and exploring the factors that influence safety, especially the views participants had about the underlying causes or factors. These stories are not necessarily considered to be objective accounts, but rather they provided an analytical window into the ways participants make sense of, give meaning to and attribute to causality to safety events. This included discussion about several vignettes, developed from the wider literature and in consultation with study advisors, and also safety situations generated by participants. This approach taken did not set out to develop objective or factual testimonies, but rather to explore the divergent perceptions, experiences and assumptions of participants. Taking this approach interviews followed a topic guide that was developed in collaboration with professional advisors, and public and patient representatives (see Fig. 1); a similar guide was used for focus groups. All interviews were recorded with the consent of participants and transcribed verbatim for the purpose of data analysis.

### Analysis

Data analysis reviewed participants’ accounts of the threats to safe discharge to identify the common and consistent safety incidents involved in hospital discharge. Although this was informed by the analytical concepts of ‘systems thinking’ [15], especially the perceptions of the active and latent factors, and the relationships between them, it is important to note that participant rarely described safety issues or events in these terms, or indeed provided well developed or linear accounts of causality. Rather, the narratives were often more ambiguous and vague [20], showing active interpretation in the context of the interview encounter [21]. As such, analysis of interview data initial aimed to elaborate three broad categories, i) safety incidents, ii) immediate proximal factors; and iii) latent distal factors. These were then re-analysed for their relationships.

In line with the above, interpretative data analysis was undertaken to develop descriptive and contextualised understanding of discharge planning and care transition across each research site. This involved an iterative process of close reading of data, coding, constant comparison, elaboration of emerging themes and re-engaging with wider literature. In the first instance, three members of the research team [JW, FM, SB] independently reviewed a sample of 5 transcripts to develop the coding strategy. At this point, one researcher [FM] took responsibility for on-going data coding and categorisation using the computer package nVivo (v.10). To assure the reliability of the coding process, codes and categories were reviewed on a weekly basis by the wider team to ensure the accuracy of interpretation and internal consistency of codes. As the coding process progressed, family codes and categories of data were identified, as well as thematic relationships.

## Results

The results section first describes stakeholders’ perceptions of safety events experienced during discharge planning and care transition. It then describes what stakeholder saw as the immediate or proximal contributory factors associated with these safety events, and finally describes the perceived distal factors that might be interpreted as the underlying root causes. The study design anticipated that stakeholders might have different experiences and perceptions of the threats to safe discharge according to the differences in the study sites (e.g. local demographics and care systems variations) and patient groups (e.g. different care needs for stroke and hip fracture patients). However, the study found relatively common and consistent findings across both the care settings and patient groups, suggesting a degree of generality, and where notable differences were identified these are highlighted below. In addition, illustrative quotes are provided within the text and a further table to quotes is provided (Table 2).

**Table 2:** Illustrative Extracts of Data

Falls	*He fell because he wanted to go to the loo. Bill shouldn’t have really gone unassisted, he did have a Zimmer frame, but he should have had a nurse,… he went off on his own.* (Relative) *I daren’t let go of the furniture you know to walk about. I need a frame or something all the time to get around. I borrow them* (Patient) *There’s always a risk of stroke patients, especially those that have gone home with a weakness of falling. There’s nothing we can do…* (Nurse)
Medicines	*We ask them if they’re still taking their bone protection medication and often they say no* (Nurse) *[I was] Given bag of medications but no instructions. No idea what they are for.* (Patient) *A lot of patients go home and for whatever reason don’t take the medicines as we have told them and experience problems* (Doctor)
Equipment	*Everything had gone in, except the mattress. The delivery man, why didn’t they just match it up, or say why are we sending this out without a mattress* (Occupational Therapist)
Infections, Sores, Ulcers	*I think a hospital is a place of safety when you’re ill and you’re brought in. There’s a saying. If you’re carried into hospital, you might walk out, but if you walk into hospital, you might be carried out. And there is an element of truth to that.* (Hospital doctor) *There is always a risk that patients will develop a pressure sore and the longer they stay in hospital with rehabilitation and without the normal activities, that risk gets bigger* (Therapist) *Hospitals are not safe places for older people. The longer they stay in, there is more chance of them picking something up. That is another reason why discharge is so important* (Hospital doctor)
Relapse	*What you really don’t want to see is the patient being re-admitted with another fracture, whether its from a fall or from a dislocation, you just don’t want it* (Doctor) *There is no certainty. You can make your best assessment and think the patient will be better cared for at home, but you can never really tell. A lot will go home and have another stroke, and we then wonder whether we pushed them out too quick or didn’t provide the necessary support* (Doctor)
Patient Assessment	*A concern is whether the patient is appropriately assessed and suitable to be discharge. The surgeon might see the patient as surgically fit, but there can be a lot of rehab and therapy input still needed. But its not always easy getting that point across* (Physiotherapist) *You sometimes get the sense that the patients are being rushed out of the door, what with all the patients coming in the front door. So we are seeing patients arrive home who are still really unwell and poorly.* (Community nurse)
Ordering Equipment & Medicines	*…every Friday … one of the doctors* *throws her hands up in horror if she has to do any TTO’s and quite often she’ll say she’s too busy, so then that means we’ve got to wait then till Monday.* (Nurse) *quite often there tends to be a day or two where the equipment isn’t in stock and it’s going to be delivered* (Physiotherapist) *we’ll fight over who orders what, who’s budget it’s going to come out of? ‘No. It’s a social commode.’ What on earth is a social commode?* (Social Worker)
Follow-up	*We see patients when they get home and we look for their care plan, and its nothing, its just a few notes about mobilisation or medicines. There is nothing detailed about what level of care they need. So we spend a lot of time re-assessing the patient and devising new care plans* (Social care) *We always try to see the patient after they get home, but we have a lot of patients and it wont be straight away. We usually rely upon the social care re-ablement teams to provide that initial general support. The GP doesn’t really get involved unless there is a problem.* (Community nurse)
Education	*We spend a lot of time with the patients providing structure rehabilitation with support for them to manage at home, but it is not always easy to get the messages across especially when the patient is very frail* (Occupational Therapist) *A lot of the burden falls on the family to provide support and they are not always available or informed about what their relative needs* (Nurse)
Planning	*I think the key thing is lack of continuity and all the stuff that centres around that, the documentation, the proper information, the Social Worker is not there that day…*(Nurse) *The MDTs are pretty poor. They are completely driven by the surgical and nursing priorities, and we go no look in to the decision* (Occupational Therapist) *I think sometimes the junior doctors get an awful lot of responsibility. They don’t know that patient but they’re expected to complete that discharge when they’ve never set eyes on that patient.* (Nurse)*.*
Referrals	*We can’t actually refer them to any outside services until they are medically fit. So until they reach a point where they’re medically fit for discharge we can’t actually do anything about referring them on to anybody until that point.* (Nurse) *It should be pretty straight forward, but each time it is different, and you never know which social worker you are after. And they keep changing any way. So it makes you think there is no continuity of care once they leave hospital* (Nurse)
Timing & Scheduling	*…if you stay in hospital longer than you should, you get a chest infection or you fall and fracture your hip and you die.* (Doctor) *From half-past five in the morning to strip my bed and I was sitting on a chair from that time till I got home. It had gone eight o’clock at night. I felt like I wanted to cry because, you know, I felt they just didn’t care.* (Patient)
Resource Constraints	*They’re closing these homes and the services are not available. We’re actually dealing with a very, I would say, an increase in need and service, but the services are not being put out there for whatever reason* (Occupational Therapist)
Organisational Pressures	…*It’s a process machine. I often think of it and I know it sounds a bit inhumane, but I think of it like a sausage factory….* (Nurse) *That’s what I’m on about with the pressure to get people out and maybe not come through the social work route because it comes out of their budget because we’re not joined-up are we with budgets* (Nurse) *Every morning a manager will come down to the ward and ask use to go through the daily discharges…pushing us to move get these one’s one or prioritise these patients. They never really look at the cases* (Nurse)

### Safety issues

Participants talked about a large number of threats to safe discharge, ranging from relatively unique and high-risk occurrence, such as a patient falling from a trolley during transfer, to more common but relatively insignificant events, such as delayed transportation. Four common safety incidents were described in relation to hospital discharge.

Patient falls were described by virtually all participants, especially physio- and occupational therapists, ward nurses, and family members. Falls were elaborated in three ways. First and most common, was patient falls at home or care setting following discharge, usually associated with frailty, ongoing recovery or lack of support or equipment. The second was in-transit falls, which occurred during the transfer from hospital, often considered in relation to problems of ordering and the quality of transportation services. The third was related to falls on the hospital ward, especially where discharge had been delayed and the patient was seen as remaining on the ward unnecessarily. Participants noted, for example, that when discharge was delayed, patients would seek to be more mobile on the ward, and at the same time there might be less routine supervision by ward staff because of the view that the patient was ‘ready to go’. In these circumstances patient falls were seen as more common.

The provision and use of medicines was another common threat to safe discharge. A prominent concern for hospital doctors, pharmacists and GPs, was medicines reconciliation following discharge, especially where information was not provided to inform the review of patients’ ongoing medicine regimen. A related concern was patient’s medicine use and adherence following discharge, especially if the patient was confused or had cognitive impairments; which was often related to the quality of instruction at the time of discharge (GPs and community pharmacists) or concern over ongoing supervision of medicines use (hospital doctors). There were also concerns amongst ward nurses and GPs about dispensing medicines prior to discharge, with several accounts of patients being discharged with incomplete or incorrect medicines, often because of the pressures on ordering and collating medicines in the period immediately prior to discharge.

A third group of safety incidents related to the patient's development of infection, ulcers or sores. These were described in two ways. First, community nurses and social care staff talked of the problems of patients being discharge home or to community setting with unresolved infection or ulceration. For example, it was suggested that some forms of wound care or chest infections would have been better managed in hospital and had caused undue disability or distress to patients. There was some recognition amongst ward nurses and hospital doctors that some patients would be discharged with unresolved infections, but usually where these were secondary to their primary condition and not significant. In contrast, these same groups also recognized that such conditions could easily develop in the community, especially where patients were not mobile and routinely monitored.

The fourth common safety incident was broadly associated with the relapse of a patient’s condition, in this case stroke or hip fracture. This was a much discussed concern for all participants. Although various factors were seen as contributing to this, including falls for hip fracture patients, or use of medicines for both groups, it was widely believed that premature discharge from hospital or discharge without an appropriate package of ongoing care were the likely causes of the patient being readmitted with the same condition. As outlined below, the factors underlying this were problems in assessing patient readiness for discharge and organizational pressures to transfer patients.

### Proximal factors

Proximal factors relate to those actions, conditions or triggers that were seen as the primary or immediate cause of a safety incident. Some include ‘active errors’ whilst others are more systemic contributory factors. These could be grouped into five categories.

The first relates to patient assessment prior to hospital discharge, especially in relation to whether the patient appropriately is assessed as ‘ready’ or ‘fit’ for discharge. Many ward based health professionals described how patients might be pronounced as ‘medically-fit’ by their doctors, despite nurses, therapists, or family members having ongoing concerns over the patient’s physical or cognitive health. This could lead to the patient being transferred home or to a new care setting while still requiring care that is not necessarily provided outside of the hospital, such as specialist rehabilitation. Inappropriate patient assessment was often cited, for example, to explain patient falls or relapse of condition. An associated problem with patient assessment was failure to diagnose or treat pre-existing or additional health problems, such as infections or sores, but also cognitive impairments and ‘safeguarding’ concerns, related to the patient’s safety outside of the hospital. Many participants based in the community setting believed that hospital based clinicians often overlooked these secondary health issues during discharge planning.

The second category of proximal factors related to the completion and reporting of necessary diagnostic tests. It was often described, for example, that due to demands or resource constraints in the hospital, some tests ordered during the processes of discharge planning would not be completed. This included blood tests for infection or CT scans. It was suggested that due to organizational demands and pressure on hospital beds it was often necessary to discharge patients without receiving these test results, and for the tests to be completed on an ‘out-patient’ basis, thereby requiring the patient to come back to the hospital. These risks were associated with the problems of infection and relapse and was seen as contributing to the problems of inappropriate or incomplete patient assessment. Less common, but also mentioned, was the ordering of tests deemed to be unnecessary by, for example, inexperienced doctors following guidelines inflexibly, thereby delaying discharge.

The third category related to the ordering, provision and management of medicines, and was seen as directly contributing to medicines-related safety issues. A very common concern raised by ward nurses and doctors was with the ordering of take out (home) medicines (TTOs). It was widely claimed that these problems related to the work of junior doctors who were usually responsible for completing prescriptions before discharge. In particular, it was commonly suggested that there are too few junior doctors on the ward, they often had significant time pressures to complete tasks, and could have limited understanding of patient medicine requirements. As such, prescribing was seen as rushed, incomplete or incorrect. There was variability between research sites in the way medicines were ordered, with hand-written prescriptions in patient records often being reported as illegible or incorrect, whereas the use of computer systems was seen as more accurate, but where there were problems with accessing the system. A further issue was the delivery and collation of patient medicines prior to discharge. There were many accounts of delayed discharge because of interruptions in either ordering or delivering medicines. On one ward it was also reported that medicines were delivered in an ‘un-sorted’ box, which required ward nurses to collate the medicines for individual patients, with the potential for errors. This reflected a wider concern about the lack of checking prior to issuing patients with medicines at the point of discharge. In addition, patients and family members reported poor levels of communication at discharge about medicines use after leaving the hospital. As such, a further concern for many participants was patient adherence to the instruction of healthcare professionals following discharge, and the needed for on-going supervision in medicine use.

The fourth category related to the ordering, provision and use of specialist equipment after discharge. These created a variety of risks, largely related to falls and mobility. A common problem for ward-based therapists concerned the ordering and funding of specialist equipment, such as beds or mattress for use in the patient’s home. Specifically, there was a complex and changing bureaucracy surrounding the ordering process for equipment, with multiple suppliers, each with different processes for online, telephone and/or fax ordering, and challenges in ensuring appropriate delivery. One prominent concern was determining how equipment would be paid for and whether it was a ‘healthcare’ or ‘social care’ responsibility, where the latter would be means-tested. The complexity of ordering meant that patients often received incomplete, incorrect or delayed equipment when at home. A further complication was the delivery and installation of equipment, from grab-rails and ramps, to complex machinery and new beds. There were several accounts of patients arriving home without having a bed delivered to an accessible ground-floor living space. In some instances patients would have to wait several days following discharge for new equipment to be fully installed raising the risk of falls, for example. As with medicines use, a further issue related to patient use of new equipment. Although ward and social care therapists provided patient instruction and support, it was described how patients would mis-use equipment or ‘*push themselves too far*’ creating the possibility for falls. However, a complicating factor was that patients would sometimes be instructed to use a model or version of equipment, such as a lifting device, that was different to the one provided in the community setting.

The fifth set of proximal factors related to the on-going support for the patient after discharge, especially follow-up care and monitoring. For hospital clinicians, re-admission was often seen as stemming from insufficient or low quality support in the community, due largely to the lack of care planning or communication of the care plan to appropriate agencies. A major concern for many participants was the limited involvement of GPs in post-discharge care, with community health and social care professionals describing GPs as largely absent from the discharge process and not pro-actively managing on-going health concerns or treatments. Some described how GPs were only involved in patient care where significant problems were identified by other staff groups, such as infections. Social care professionals and family members described how community nurses provided only limited support and too often patient wounds or other healthcare issues were poorly managed. In general, there was a widespread concern that patients could leave hospital with very patchy or incomplete on-going care, which exacerbated the potential for harm. As outlined below, this often related to deficiencies in care planning.

### Distal factors

Distal factors include those underlying or system level issues that participants commonly described in the course of explaining why a safety event might have occurred, and typically saw as an enduring or cross-cutting issue that impacted upon care quality through shaping the context in which more proximal factors occurred. In relation to hospital discharge six categories of factors were identified.

The first was broadly related to discharge and care planning. Although policies promote discharge planning processes and toolkits [1], the study found no common approach across the hospital sites or patient groups. There was a broadly similar planning process, comprising weekly team meetings, referral processes and transitions, but these were organised in different ways, such as the types of information shared, the timing of interactions, and the expectations around professional involvement. For example, in one locality social workers visited the hospital ward on a daily basis to discuss patient care plans, whereas for another they held ad-hoc visits only when deemed absolutely necessary, more commonly working via telephone consultation. Similarly, on some hospital wards discharge planning was undertaken during normal working hours where it was possible to contact relevant stakeholders, whereas on others it took place at night meaning many coordinating tasks were delayed until the next day, such as liaising with external agencies or obtaining additional information. As such, there was a general sense of complexity, confusion and poor integration between the different health and social care systems of work that was seen as conditioning many of the problems of care planning.

More specific problems with discharge planning were associated with the involvements of relevant stakeholders, especially in weekly meetings and daily activities. For example, social workers described how it was difficult to coordinate their work to attend planning meetings organised around ward-based clinical schedules. Similar issues were described by therapists who found it difficult to attend some planning meetings due to clinical duties in other areas. Therapists and some nurses also described how their involvement in care planning was constrained due to decision-making being dominated by more medical issues. This meant that concerns about patient mobility, cognition or lifestyle factors were not fully considered, and the decision to escalate discharge was taken on the grounds of being ‘medically-fit’. In all but one hospital setting, participants described difficulties of getting their voice heard and making a contribution to care planning, because of underlying professional hierarchies and the pressures to expedite hospital discharge. As a consequence of the problems in discharge planning, most participants suggested that on-going discharge plans would often be incomplete or missing important detail.

The second group of distal factors also related to the process of discharge planning and centred on making referrals and communicating with external agencies during or after discharge. An important activity in discharge planning is when hospital staff contact community health and social care professionals to initiate onward care planning. Many common problems were experienced with these referral processes, including determining whether a patient needed (and was eligible for) health and/or social care, completing the necessary forms and paperwork, identifying the appropriate point of contact outside of the hospital, and scheduling a referral visit or telephone conversation. A large variety of complications were identified with these steps, which often resulted in delays in the planning process or patients not being provided with an appropriate care plan. For example, ward nurses described problems with the social care system’s ‘single point of contact’ or telephone centre, which made it difficult to identify or locate a given (named) social worker involved in a on-going care plan. As well as problems in contacting external groups, ward staff also described problems in accessing relevant patient information when making referrals, especially where patient information was distributed in different medical, nursing and therapist record systems, rather than in a single ‘shared record’.

A further problem in the relationship between hospital and community care providers was the communication of the patients care records and onward care plan at the point of discharge, including lost information on medicines, on-going care needs and rehabilitation. Despite the acute sites installing electronic systems, there was a large variation in the forms of communication accepted by ongoing care agencies. For example, many GPs relied on traditional ‘discharge letters’ summarising hospital care and on-going care needs. These often arrived with the GP many days after discharge or contained limited information. Similarly, community hospitals, community care teams and social care agencies requested the completion of different forms, care summaries or telephone questionnaires, causing confusion and frustration amongst ward staff. Recipients of these referrals described a major problem with missing information and needing to complete new assessment procedures with patients upon arriving in the community to ensure an appropriate care plan was in place. As such, the on-going care plan was often made and re-made through a series of initial meetings because of the failure to thoroughly plan and communicate care needs prior to discharge.

A third distal factor, also stemming from the planning process, related to the timing and scheduling of discharge. Two common aspects of timing were described, either premature or delayed discharge. Premature discharge was seen as problematic because patients left hospital not fully recovered from their condition or without the necessary care or equipment in place for their on-going recovery. This was associated with organisational pressures to discharge patients and turnover hospital beds. In contrast delayed discharge was seen as creating risk factors because of the impact it could have on the wider arrangement of care, for example complicating the start of planned home-care or rehabilitation. Delayed discharge was also associated with creating additional risks to hospital-acquired infection or dependencies on care staff. As such, many participants talked of an ideal ‘window of opportunity’.

A further timing issue was the general mis-match of scheduling between hospital and community care providers. It was described, for example, that hospital clinicians typically arranged discharge for the end of the day and week, thereby allowing the completion of tests and planning, but that social care providers sought to commence work with new patients at the beginning of the day and week. As such, a common phenomenon was for patients to be discharged home on Friday afternoon with only limited social care provision until Monday morning, which was often seen as exacerbating the potential for fall or relapse in the immediate post-hospital period.

A fourth category of factors relates to the profile of resources within each care system. A particular problem for participants in Health System 1, was the relatively limited availability of community hospitals or intermediate care services. As such, patients leaving hospital were required to either return home with a package of care, or enter a private and local authority residential care facilities, with funding through the local health or social care commissioners. According to participants this limited the range of options for discharge planning with patients often having sub-optimal care plans. It also seemed to create extra pressures on these available services with difficulty in accessing residential homes or having limited availability for home-care services. This could therefore create additional delays in discharge or mean patients were not provided with appropriate levels of follow-on care. In contrast, Health System 2 had two community hospitals that provided step-down and intermediate care services. This made it possible for hospital staff to plan and transfer care more easily outside of the acute setting, but it also created additional pressures on these community hospitals as they saw themselves as the ‘over-flows’ for the acute hospital and faced additional pressures of planning future care. As such, they described a situation in which responsibility and risk was transferred along with the patient. A more general concern for both areas was the availability of social care services given recent funding pressures, with fears that care packages and the range of available services were being reduced to meet budgetary cuts.

The final and related category of distal factors concerned the managerial pressures and cultural expectations of the different care organisations with regards to hospital discharge. For the acute hospitals, the transfer of patients was described as a priority because it made available inpatient beds and enabled greater ‘flow’ of patients through the acute sector. Moreover, prolonged admission was seen as a costly waste of resources. Ward staff described a constant pressure to expedite discharge, creating the possibility for patients to be discharge prematurely, for plans to be incomplete or for tasks to be rushed. Hospital staff also talked of similar pressures in the local social care setting, where similar resource constraints had limited the availability of care services, especially residential care. Supporting this, some social workers also suggested that their organisational environment was designed to limit care provision to as little as could be justified, rather than as much as necessary for optimal recovery. This was seen as making it frequently difficult to find suitable care arrangements as care responsibility was ‘worked out’, which many saw as underlying the problem of delayed discharge.

## Discussion

Hospital discharge is a vulnerable and high-risk stage in the patient journey, requiring the interaction and coordination of multiple health and social care professionals and organisations. It is the complexity of these interactions that appears to underscore the quality and safety of patient care. Through exploring the experiences and perceptions of different stakeholders, this complexity is brought to light and through these accounts it is possible to better understand the different threats to safe hospital discharge, and what stakeholders understand as the factors that condition or bring about safety threats and the relationships between them [12]. Through better recognising these perceptions and viewpoints, it becomes possible to develop and target interventions that address the concerns of frontline clinicians.

Patient safety research has been influenced greatly by theories and models within the ergonomics and human factors to encourage greater analysis of the relationship between ‘active’ errors in frontline practice, and ‘latent’ factors found in the work environment, organization or care system [15]. Although this is a powerful analytical framework for safety scientists, it did not easily map onto the descriptive and explanatory narratives of participants. Participants appeared to provide highly detailed and specific illustrations of safety events, but they rarely categorised these incident in terms of specific errors or contributory factors. Moreover, participants rarely offered clear or linear casual accounts, e.g. ‘A led to B which led to C’ , rather they developed more complicated and non-linear accounts; discussed further below. In seeking to reflect the more complex ways in which participants talked of the threats to safe discharge, the approach taken in this study was to analyse and categorise accounts in terms of broader, and less pre-determined, ‘proximal’ and ‘distal’ factors, which allowed for more open interpretation of the assumed causal relationships underpinning respondents accounts.

**Fig. 2:**
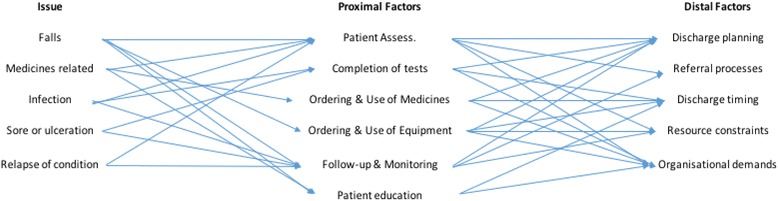
Analysis of relationships between factors

Looking closer at the categories described, and the relationships between them, the study identifies a number of prominent assumed casual relationships that might be the focus of further research and intervention (see Fig. 2). Although each of the identified proximal factors clearly has an important bearing on different aspects of safety, for example the ordering of medicines has a bearing of medicines safety, the analysis suggests ‘patient assessment’ and ‘follow-up and monitoring’ was seen more often, and by more stakeholders, as having a bearing on discharge safety. Specifically, patient assessment was seen in various ways as contributing to all prominent safety issues, and was associated with other proximal risks, such as ordering of equipment or medicines. Similarly, follow-up and monitoring of the patient after discharge was associated with common safety issues. These might therefore provide two core activities or priorities for safety enhancement.

Turning to the wider or underlying distal factors, it was more difficult to detect clear or direct causal assumptions, but a number of common risk factors were described. In particular, these highlight the importance of discharge planning, especially the involvement of all relevant stakeholders in decision-making, the timing or planning activities, and the completion of tasks allocated during planning. Linked to this were similar problems with external referrals. Together these planning and communication activities were seen to complicate or condition various proximal factors, especially the provision of follow-up care, which is largely dependent upon the quality of planning and accuracy of referrals. It is also the case that the completion of patient tests and the quality of assessment could be influenced by the planning process, such as the inclusion of relevant clinical perspectives. Yet, this is also a two-way relationship as failure to complete assessment or tests invariably undermine discharge planning processes. The other commonly described distal factor was organisational pressure, linked to resource constraints. It was described how these created the conditions, through cultural or managerial expectations, in which discharge planning might be pressurised, and therefore incomplete, partial or substandard. Similarly, patient assessment, patient education, and ordering of equipment and medicines were complicated by organisational pressures. As such, the views of different stakeholders suggest greater attention to the quality of discharge planning (format, scheduling, involvement) in the context of wider organisational pressures might be key priorities for safety enhancements.

Reflecting further on the study findings, the views of participants reinforce the importance of communication in the processes of discharge planning and care transition. The wider literature on hospital discharge repeatedly suggests that effective and timely communication can reduce system complexity and support coordination, for example, in discharge planning or the use of checklists [4, 5, 8, 9, 12, 14, 22]. The wider literature on communication highlights a range of facilitating or inhibiting factors, ranging from the form and structure of language or knowledge being communicated to the characteristics of both ‘donor’ and ‘recipient actors’, such as their motivations, accessibility, levels of trust, values, hierarchies and absorptive capacity. Similarly, power hierarchies and cultural difference between actors can impact communication [2, 23]. As such, efforts to promote communication and coordination at the areas identified above might be priorities for safety improvements.

It is important, however, to be cautious in the use of participant accounts alone in the analysis of patient safety. As noted above, participants rarely provided clear or unambiguous safety narratives, rather they highlighted uncertainty and ambiguity in perception and understanding of the threats to safety [20]. In many instances participants provided a number of highly detailed and developed accounts of safety incidents, but when asked about risk factors in more abstract terms they would struggle to give an illustration of the consequences, for example, of an incomplete discharge plan. This might be explained by the difficulty that stakeholders have in understanding activities or conditions that occur in care settings other than their own. So for example, it was difficult for community social care providers to describe in detail why equipment might be missing, but they would infer or assume it was related to either the equipment supply or ordering processes. Similarly, those in the hospital environment had limited appreciation of the safety events that transpired in the community, such as the significance of an undiagnosed infection or ulcer. In other words, participants' understanding of discharge safety was shaped by their distinct position within the care process and the 'sight-line' or scope of perception this afforded for understanding either the preceeding causes of an event, or the consequences of their actions.  As such, the way in which stakeholders experienced, perceived and made sense of safety issues rarely involved linear chains of causality, rather they could be seen as reflecting assumptions prevalent in one part of the health and social care system. Moreover, these assumptions could be seen to reflect implicit and explicit forms of inter-group blame [24], or the tendency to allocate responsibility to other actors in the care system. For example, social care professionals often found fault with hospital staff or GPs, similarly ward nurses and therapists often criticised the way in which doctors dominated decision-making. These aspects of participants’ accounts reveal cultural differences and tensions between stakeholder groups, and the influence of politics and power in shaping the integration and coordination of care at the interface of the hospital and the wider care system. In other words, participants’ understanding of the threats to safety might be seen as reflecting and perpetuating underlying tensions and disagreements between professional groups about how care should be organised, and how certain groups create risks that undermine patient safety. As such, it is important to caution against using stakeholder perceptions and accounts directly without considering these psychological, cultural and political dimensions. Moreover, cultural and political dimensions might also be seen as an underlying social threat to safety that exacerbates many of the identified complications and risk factors. In other words, the accounts examined in this study provide insight into discharge risks, processes and cultures in their own right, while the assumed causal relationships identified provide the basis for future empirical study.

### Study limitations

It is important to reflect upon the limitations of the study. Building upon the previous section, the paper relies primarily upon participant accounts of their experiences and perceptions of hospital discharge, and as such, might be interpreted as inherently subjective, imbued with bias and culturally framed. Rather than seeking to control for or limit such bias, the study was explicitly open to and considered such issues, as interpreted these within the context of their particular social and cultural setting. There is scope, however, to draw upon additional data sources that might provide additional data to challenge or substantiate the views of participants, such as observations and documentary analysis.

It is also important to note that the study was undertaken in two particular care systems with two defined patient groups. As noted above, it was anticipated when designing the study that there would be significant differences both between care settings and patient groups, but the study found relatively common views amongst stakeholders. It remains important, however, to caution against over-generalisation from the study findings. Although the study sites reflected relatively typical urban acute settings albeit with different urban and rural demographics, they are not necessarily representative of the entire English health and social care or indeed international care systems. For example, it might be expected that large urban metropolitan areas or more dispersed rural locations would represent different care settings with distinct issues for hospital discharge. Similarly, stroke and hip fracture patients were selected because they are often elderly patients with complex care needs, but it is important not to assume that the issues associated with these patients are typical of other patient groups, or care transitions. It might be expected that patients with less complex health and social care issues would experience less protracted discharge planning, or indeed those with relatively distinct care needs, such as children or cancer patients, would face distinct challenges.

A final point for consideration is that the study was undertaken within the English health and social care systems at a time of reform and where both systems faced considerable financial constraints. Specifically, the English NHS was experiencing a further re-organisation in its commissioning arrangements, and there were considerable budgetary pressures within the social care sector. As such, it is important to consider how such changes and constraints were influencing the views of participants, and potential exacerbating their perceptions of risk.

### Recommendations

Based upon the experiences and perceptions of stakeholders, together with the analysis presented above, the paper offers some tentative recommendations for how discharge planning and care transition might be improved to promote patient safety. Building on previous work [23] the authors suggest that three aspects of discharge planning might be the focus for interventions to the promote discharge safety. First, is the need to enhance discharge planning and coordination processes through greater involvement of relevant stakeholders. Existing discharge planning frameworks almost universally recommend the importance of shared and multi-disciplinary decision-making [1–3], and this study similarly finds widespread support for more inclusive forms of care planning. However, this might involve more than fixed weekly meetings, which are often difficult for some non-hospital stakeholders to attend, and instead involve meetings that are either scheduled at different times of the day or week, or make use of available information computer technologies to facilitate remote access. It is especially important that stakeholders located outside the hospital are more directly involved in the planning of care to be provided following discharge.

Second, the study also supports calls for more streamlined and shared forms of communication and information exchange [2–4]. Stakeholders raised concerns about the use of different information and communication systems, including the duplication of information, the variability of information quality, and the timing of information exchange. With growing use of digital and computer technologies there might be scope to determine commonly agreed standards for communication as well as locally determine frameworks for communication expectations. More specifically, the development of common or shared information systems that are used across stakeholder groups might facilitate collaboration and communication.

Third, the study suggests the responsibility for hospital discharge is often dispersed and fragmented within the care system, often being ‘handed-over’ as the patient moves between settings. Hospital discharge not only represents the physical transition of the patient, but also the transfer of professional responsibility for the patient. This might explain why stakeholders did not always feel compelled to engage in processes or activities that were outside of their sphere of responsibility, and hence this might explain the problems of coordinating care. It might be recommended therefore that stakeholders share responsibility for care transitions, where external (community) agencies are as equally responsible for discharge planning that takes places in the hospital, as hospital staff are responsible for assuring on-going care delivery in the community. This might involve developing shared governance procedures or even legal obligations for shared accountability.

Finally, the study suggests there is greater opportunity for patients, and their relatives and carers, to play a greater role in discharge planning and care transitions. Significantly, the patient is often the only point of continuity and consistency across the complex care pathway, and where other professionals and carers will play different parts, the patient remains. As such, the patient and their relatives can be a useful point of reference and medium for communication and knowledge exchange, although it is also acknowledged that patients with complex care needs and cognitive impairments might not be well suited to fulfil this role. Nevertheless, patients and their relatives could have an important and influential role in planning processes and opportunities might be sought to more fully involve and integrate patients in decision-making activities.

## Conclusion

Hospital discharge is widely accepted as a high risk and vulnerable stage in the patient journey. With comparatively limited research on this care process, this paper investigated stakeholder perceptions and narratives about the threats to safe discharge. It looked, in particular, at the different types and categorise of risk and the assumed causality, in terms of proximal and distal factors. The study identified: (1) ‘direct’ patient harms comprising falls, infection, sores and ulceration, medicines-related issues, and relapse; (2) proximal ‘contributing’ factors including completing of tests, assessment of patient, management of equipment and medicines, care plan, follow-up care and patient education; and (3) distal ‘latent’ factors including discharge planning, referral processes, discharge timing, resources constraints, and organisational demands. Looking at the assumed relationships between these factors, stakeholder perspectives suggest patient assessment in hospital and follow-up and monitoring in the community setting were the main common immediate causal concerns of participants, with problems with care planning, referrals and organisational pressures as being the underlying factors. Focusing on these key areas might represent optimal areas for management or service intervention to promote patient safety during hospital discharge.

## Abbreviations

GP, General Practitioner; HCA, Health Care Assistant; NHS, National Health Service; NIHR, National Institute for Health Research.
